# Statistical Thermodynamic Description of Self-Assembly of Large Inclusions in Biological Membranes

**DOI:** 10.3390/cimb46100643

**Published:** 2024-09-26

**Authors:** Andres De Virgiliis, Ariel Meyra, Alina Ciach

**Affiliations:** 1 Instituto de Física de Líquidos y Sistemas Bilógicos, Facultad de Ciencias Exactas-UNLP-CONICET, La Plata 1900, Argentina; adevir@iflysib.unlp.edu.ar (A.D.V.); agmeyra@gmail.com (A.M.); 2 Departamento de Ciencias Básicas, Facultad de Ingeniería, Universidad Nacional de La Plata, La Plata 1900, Argentina; 3 Departamento de Ingeniería Mecánica, Facultad Regional La Plata, Universidad Tecnológica Nacional, La Plata 1900, Argentina; 4 Institute of Physical Chemistry, Polish Academy of Sciences, 01-224 Warsaw, Poland

**Keywords:** membrane proteins, spontaneous pattern formation, inhomogeneous mixtures, self-assembled patterns

## Abstract

Recent studies revealed anomalous underscreening in concentrated electrolytes, and we suggest that the underscreened electrostatic forces between membrane proteins play a significant role in the process of self-assembly. In this work, we assumed that the underscreened electrostatic forces compete with the thermodynamic Casimir forces induced by concentration fluctuations in the lipid bilayer, and developed a simplified model for a binary mixture of oppositely charged membrane proteins with different preference to liquid-ordered and liquid-disordered domains in the membrane. In the model, like macromolecules interact with short-range Casimir attraction and long-range electrostatic repulsion, and the cross-interaction is of the opposite sign. We determine energetically favored patterns in a system in equilibrium with a bulk reservoir of the macromolecules. Different patterns consisting of clusters and stripes of the two components and of vacancies are energetically favorable for different values of the chemical potentials. Effects of thermal flutuations at low temperature are studied using Monte Carlo simulations in grand canonical and canonical ensembles. For fixed numbers of the macromolecules, a single two-component cluster with a regular pattern coexists with dispersed small one-component clusters, and the number of small clusters depends on the ratio of the numbers of the molecules of the two components. Our results show that the pattern formation is controlled by the shape of the interactions, the density of the proteins, and the proportion of the components.

## 1. Introduction

Plasma membranes in live cells are quasi-two-dimensional liquid mixtures of different lipids, cholesterol and transmembrane or anchored proteins. It is already well established that at the macroscopic length scale, the membranes in live cells are homogeneous, but at the mesoscale, which is larger than the range of the direct interactions between the membrane components, transient domains resembling liquid-ordered (Lo) and liquid disordered (Ld) phases appear [1,2,3,4,5,6,7]. Numerous studies have confirmed that this heterogeneity follows from the fact that the thermodynamic state of the plasma membrane in live cells is close to the miscibility critical point [2,6]. Below the miscibility critical temperature, the separation into Lo and Ld phases would take place, and indeed, this was observed in membranes extracted from cells at temperatures lower than the temperature of the growing cell [6,7]. In the phase with mixed components, the size of the domains with the local composition different from the average composition increases when the thermodynamic state approaches the miscibility critical point, and depends on the ratio of the thermodynamic parameters to their critical values, not on the details of the interactions. For this reason, simplified models can be used to describe the universal critical phenomena [3,6,8].

According to recent studies, the heterogeneity in lipid membranes plays an important role in biological functions [6]. Some proteins are preferably soluble in the Lo phase, while other ones in the Ld phase, and the functions of many proteins depend on the local environment [7]. When the Lo or Ld domains formed around certain membrane inclusions start to overlap, they induce effective interactions between the inclusions that are attractive or repulsive for the domains of the same or of different type, respectively [6]. Such interactions are universal, i.e., are present between any selective objects immersed in any solvent that is close to the miscibility critical point, and are called the thermodynamic Casimir potential [2,8,9,10,11,12,13,14]. In the following, we use the term ’Casimir potential’ for the thermodynamic Casimir potential for simplicity of notation. However, the thermodynamic Casimir potential resulting from the confinement of density or concentration fluctuations near the critical point in fluids should not be mistaken with the original Casimir potential resulting from the confinement of quantum fluctuations.

The clustering of like proteins can be enhanced by the presence of ligands or antibodies, and the local concentration around the cluster differs from the average concentration in the whole membrane [7]. In this context, the theoretical modeling of these highly dynamic compositional microdomains has remained quite challenging, since the mechanisms that regulate the shape, size, and also the lifetime of the spatially organized regions are influenced by lipid–lipid and lipid–protein interactions [5,15].

In general, attractive interactions of any origin lead to a macroscopic separation of dilute and dense phases at sufficiently low temperature. Effective attractions between the proteins, however, lead to the formation of clusters in different parts of the membrane, but a single droplet of the phase rich in proteins is not formed. One can argue that live cells are not in thermal equilibrium, and the coalescence of the clusters would take place on a larger time scale. Several other mechanisms that would inhibit further growth of the self-assembled clusters have been suggested [6], but the issue remains controversial.

In this work, we suggest another possible reason for the inhibited further growth and coalescence of the clusters of membrane proteins. Macromolecules in cells are charged, and the electrostatic repulsion between proteins with like charges should counteract the aggregation. However, the ionic cloud around a charged object screens its charge. According to classical theories [16,17], the screening length (the size of the neutralizing ionic cloud) decreases with an increasing density of ions. Since the concentration of ions such as Na^+^, K^+^, Ca^2+^, and Cl^−^ around macromolecules in cells is very large [18], it is natural to assume that the screening length is much shorter than the range of the effective attractive interactions, and the screened electrostatic interactions can be neglected. Recently, however, it was discovered that in concentrated electrolytes, beyond about 0.5 M, the screening length starts to increase with an increasing density of ions [19,20,21,22]. The anomalous underscreening was experimentally observed in a number of concentrated electrolytes and studied by theory and simulations [22,23,24,25,26,27,28,29,30]. The range of the electrostatic interactions between charged objects in concentrated electrolytes is very large; for example, the force between charged mica cylinders immersed in the 2 M NaCl_(aq)_ was measured up to 6 nm [19]. The magnitude of these interactions at large distances, however, is small [22]. The underscreening can be explained by ionic association into charge-neutral dimers or larger aggregates by which there remain fewer free ions capable of neutralizing the charged objects. The association becomes more efficient with an increasing density of ions, and the larger the density of ions, the less free ions remain in the system [30,31]. Importantly, the anomalous underscreening is universal in the sense that the screening length is proportional to the product of the density of ions and the Bjerrum length in various concentrated electrolytes [20,25]. The density of ions around macromolecules in live cells is very large—around DNA, it can be as large as 10 M [18], therefore similar underscreening can be present in the case of charged membrane inclusions surrounded by a dense ionic cloud.

If the hypothesis of anomalous underscreening in live cells is correct, there are two universal interactions between charged membrane inclusions with preferential solubility in one type of lipids, namely the Casimir and the underscreened electrostatic interactions. Notably, these interactions are of the *opposite* sign. Like proteins attract and repel each other with the Casimir and the electrostatic potential, respectively. In contrast, the Casimir and the electrostatic potentials between oppositely charged proteins with different preferential solubility are repulsive and attractive, respectively.

Let us consider the effect of the competition between these two types of interactions on a general level. The total interaction between charged selective objects depends on the ranges and strengths of the attractive and repulsive contributions to the potential. Let us first assume that the range of the Casimir potential is larger, but its magnitude is smaller. In this case, the total interaction takes a minimum at a rather large distance, and the overal shape of the potential resembles the shape of the interactions between atoms or molecules [32]. In this case, the macroscopic separation of dilute and dense phases should be expected. Indeed, experiments for colloidal particles in near-critical water–lutidine mixture have shown the formation of dense droplets in three-dimensional space upon approaching the critical temperature of the solvent [12].

If the range of the underscreened electrostatic interactions is larger and its amplitude is smaller, the resulting potential between like proteins may have the form of short-range attraction and long-range repulsion (SALR) [32]. Such interactions with different shapes and ranges of the attractive and repulsive parts have already been investigated in two-dimensional (2D) or quasi-2D systems [13,33,34,35,36,37], and in all cases, the formation of almost monodisperse clusters was observed at low densities and relatively high temperatures. At low temperatures, a dilute dispersion of particles is observed at very low density, and for increasing density, the sequence of stable phases is: clusters forming a hexagonal lattice, stripes, liquid with hexagonally ordered bubbles, and finally dense liquid. Only the size of the aggregates changes upon variation of the shape of the interactions. The stripe and hexagonal arrangements of domains and transitions between them were observed in a vesicle adhering to a supported lipid bilayer [38], in agreement with theoretical predictions for ordered patterns [33,34,35,36] .

Since in concentrated ionic solutions the magnitude of the underscreened electrostatic interactions is small and its range is very large, we may expect that the charged membrane inclusions preferring one type of lipid may interact with the SALR potential if the local concentration of ions is well above 1M. Note that this hypothesis is consistent with the formation of almost monodisperse protein clusters surrounded by the preferred domains in the membranes. Of course, further studies should clarify if the effective interactions between certain membrane proteins in certain conditions indeed have the SALR form. Experimental studies on charged inclusions with preferential solubility in one type of lipid in a near-critical lipid bilayer surrounded by concentrated ionic solution should shed more light on this issue.

The self-assembly of the particles interacting with the effective SALR potential can be significantly influenced by the presence of oppositely charged membrane inclusions. Instead of focusing on particular cases, we ask again a very general question about spontaneous pattern formation in a mixture of oppositely charged particles or macromolecules with a different preferential solubility in components of a quasi-2D critical liquid mixture that, in turn, is located in a concentrated ionic solution. Our main question is what types of patterns are energetically favorable for different strengths and ranges of the interactions between like and different particles. Based on the properties of one-component SALR systems, we expect that the ordered low-temperature patterns lose their long-range order upon heating stepwise. First, the periodic spacial distribution of the aggregates is destroyed, but the short-range order is maintained, i.e., the aggregates do not disintegrate and become mobile. Further heating leads to the random distribution of the particles or molecules.

The mixture of self-assembling particles or molecules has attracted increasing attention [39,40,41,42,43,44,45], but the question of how the self-assembled patterns depend both on the relative ranges and strengths of the attractive and repulsive parts of the potential and on the concentration of species remains open.

It should be mentioned that there may be many other sources of the SALR potential in biological and soft-matter systems, including the basic DLVO potential that is also relevant to protein interactions [15]. In addition, interactions other than the SALR potential can lead to microsegregation if there is a competition between two length scales in the pair potential, such as, for instance, with core-softened potentials with attractive cross-interaction [46,47] or in the case of competing amphiphilic and solvophobic interactions [48,49]. Thus, studies on pattern formation in mixtures with competing interactions may concern a large class of systems with mesoscopic inhomogeneity.

Pattern formation by adsorbed particles can be affected by the curvature of the surface, as shown for example, for adsorption on a sphere [50,51]. In this work, we limited ourselves to large biological membranes compared to the size of the aggregates, and we considered the triangular lattice model introduced and studied in Refs. [43,44]. Previous studies focused on the interactions between like particles with repulsion at large distances being significantly stronger than the attraction at short distances. In addition to the patterns present in a one-component system, the alternating stripes of two components, parallel chains of alternating clusters, zig-zag patterns, and clusters of one component filling holes in a second component were obtained for some regions of the plane of chemical potentials (or densities of the two components) [43,45].

For the present work, we assume that the repulsive part of the potential is weaker than the attractive part. In the case of one kind of self-assembling particles, it was observed that weaker repulsion at large distances leads to larger clusters, but otherwise, the sequence of ordered patterns remained the same [35]. Now, we address the question of if the same phases are present in the mixture for different shapes of the interactions, or if new patterns can emerge when the relative strength of the repulsive and attractive parts of the potential is varied. We find that the pattern formation is much more complex, and different sequences of ordered patterns are obtained when the interaction at large distances is weakened but the range remains unchanged. This result is in a strong contrast to the one-component case, where the universal pattern formation was found in 2D and 3D cases [33,34,35,36].

In Section 2, we introduce the model and describe the simulation method. The results are presented in Section 3. In Section 3.1 and Section 3.2, the energetically favorable patterns in an open system that exchanges molecules with the bulk reservoir are shown. The results presented in Section 3.1 were obtained by theoretical calculations. The effects of thermal motion at low temperature (kinetic energy much smaller than the potential energy) are obtained by GCMC simulations and discussed in Section 3.2. Finally, Section 3.3 presents the simulation results for fixed numbers of particles in the canonical ensemble for different proportions of the first and the second component. Section 4 contains the summary and conclusions of this paper.

## 2. Model and Simulation Method

Biological membranes are three-dimensional (3D) objects, but their thickness in the direction perpendicular to the lipid bilayer is comparable with the size of the membrane proteins, and the patterns are formed in the lateral directions. Thus, the membranes can be considered quasi-2D objects. A simplified model of a quasi-2D object is, for example, a mixture confined in a slit with a thickness that is a bit larger than the thickness of the largest molecules. Two-dimensional and quasi-two-dimensional models of self-assembling systems have been developed and studied, and in all cases, the same qualitative behavior concerning pattern formation was obtained [13,33,34,35,36,37,45]. Thus, we assume that for pattern formation by transmembrane proteins or other membrane inclusions, the third dimension plays a subdominant role and 2D models predict the lateral patterns sufficiently well. In one of these 2D models [35,36], the particles occupied the cells of a triangular lattice for the simplicity of simulation, but this approximation did not alter the sequence of spontaneously formed patterns. A similar lattice model was introduced for the mixture of two-types of SALR particles with cross-interaction of opposite sign [43].

**Figure 1:**
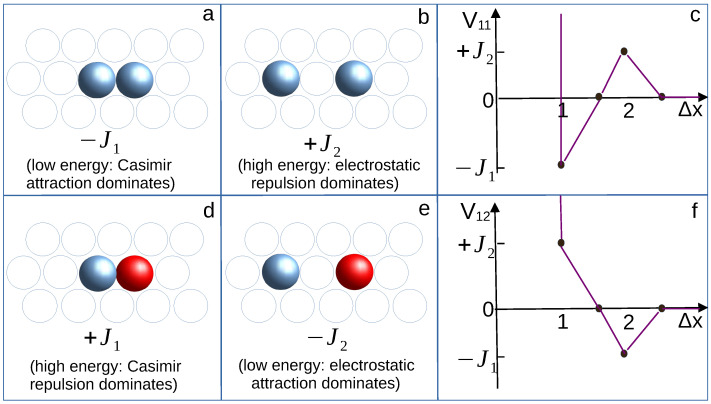
Figure showing the cells of the triangular lattice filled with two like particles (panels (**a**,**b**)) and with two different ones (panels (**d**,**e**)). The interaction energy for each pair is indicated, and $V_{ij}$ is shown by the symbols in panels (**c**,**f**) for like and different particles, respectively; the lines are to guide the eye. Red and blue spheres represent particles of the first and the second component, respectively. The interaction between two second-component particles (blue spheres) is the same as the interaction between two first-component particles (red spheres), i.e., $V_{22}=V_{11}$. All pairs of particles separated by the distance |Δx| give the same contribution to the energy of the system, independently of the direction of Δx.

Following ref. [43], we assume that the particles or macromolecules occupy cells of a triangular lattice, and each cell can contain no more than one particle. The linear size of the cell, equal to the diameter of the particles, is taken as the length unit (Figure 1). The particles in the cells separated by the vector Δx connecting the centers of the cells interact with the potential (see Figure 1)

(1) $$V_{ij}\left(\Delta x\right)=\left\{\begin{array}{c} V(\Delta x)\;\mathrm{for}i=j\;\\ -V(\Delta x)\;\mathrm{for}i\neq j\; \end{array}\right.$$

where i,j=1,2 refer to the first and the second component, and

(2) $$V\left(\Delta x\right)=\left\{\begin{array}{c} -J_{1}\;\mathrm{for}|\Delta x|=1,\;(\mathrm{for}\mathrm{nearest}\mathrm{neighbors})\\ +J_{2}\;\mathrm{for}|\Delta x|=2,\;(\mathrm{for}\mathrm{third}\mathrm{neighbors})\\ 0\;\mathrm{otherwise} \end{array}\right.$$

where $-J_{1}$ and $J_{2}$ are the energies of the attraction and repulsion between like particles for |Δx|=1 and |Δx|=2, respectively, and the cross-interaction is of the opposite sign. The lattice model defined above has the key features of a binary mixture of oppositely charged particles or macromolecules with different solubility in a mixture close to the miscibility critical point.

The energy of a particular distribution of particles is equal to the sum of energies of all interacting pairs, and is given by the formula

(3) $$E=\frac{1}{2}\sum_{x}\sum_{x^{\prime }}\rho_{i}\left(x\right)V_{ij}\left(x-x^{\prime }\right)\rho_{j}\left(x^{\prime }\right),$$

where $\rho_{i}\left(x\right)=1$ or 0 if the cell x is occupied by the particle of the *i*-th component or not, respectively. The sum $\sum_{x}$ is made over all lattice cells, and summation convention for repeated indexes is used. If the quasi-2D liquid containing the particles (or macromolecules) is allowed to exchange particles with a bulk reservoir, there is an additional contribution to the grand thermodynamic potential equal to the chemical potential $\mu_{i}$ that is associated with an insertion of a particle of type *i* to the system. In this case, we consider the thermodynamic Hamiltonian

(4) $$H=E-\mu_{i}\sum_{x}\rho_{i}\left(x\right)$$

where $\sum_{x}\rho_{i}\left(x\right)$ is equal to the number of particles of the *i*-th component. We choose $J_{1}$ for the energy unit, and define the parameter $J=J_{2}/J_{1}$. The chemical potentials will be measured in $J_{1}$ units as well.

The most probable distribution of the particles, corresponding to the minimum of *E* or *H* for a fixed number of particles or for equilibrium with the reservoir, respectively, is not destroyed by thermal motion at T=0. If the energy of thermal motion is $k_{B}T\ll J_{1}$ with $k_{B}$ denoting the Boltzmann constant and *T* the temperature, the potential energy dominates over the kinetic energy, and the minimum of *E* or *H* gives a fair approximation of the equilibrium structure. For increasing temperature, the competition between the entropy and the energy leads first to an increasing number of defects, next ordered domains with different orientation appear, and finally, a random distribution of the particles is present at very high *T*. Before analyzing the more complex case of $k_{B}T∼J_{1}$, one should determine those ordered patterns that are energetically favored in the first place, and this is the purpose of this paper.

In our previous studies, we assumed $J_{2}=3J_{1}$, since this case was thoroughly studied for the one-component system [35,36]. In the present work, we considered the ratio $J=J_{2}/J_{1}=3/4$ to determine how the strength of like-particle repulsion for |Δx|=2 influences the pattern formation. Now, one can expect a more complex scenario for the phase portrait of the mixture, since this relatively low repulsion might allow for a subtle competition between clustering and phase separation processes.

We follow the same strategy as in Ref. [43]. The types of ordered patterns are found by Monte Carlo simulations at finite (but sufficiently low) temperatures for several points in the $\left(\mu_{1},\mu_{2}\right)$ plane, and *H* was calculated for the unit cell of each periodic pattern using the periodic boundary conditions. The stability regions of different phases were determined by comparison with the thermodynamic Hamiltonian per lattice cell in the ordered phases. One advantage of the simulation methodology is that it provides information about the role of thermal fluctuations on the phase ordering of the mixture. In this context, in future studies, we will explore the evolution of the ordered patterns with temperature in some specific cases of interest.

The simulation procedure was described in detail in Ref. [43]. In this scheme, we fix the temperature at low values and then produce scans in the chemical potential $\mu_{i}$ of each species (*i* = 1,2), applying the parallel tempering method. This strategy involves running simulations in parallel for different samples of the system, each of them under fixed values of chemical potentials that are assigned after binning the interval of interest for the μ’s. Then, starting from a lattice occupied at random by both species, particles are inserted or removed with probabilities that follow the Metropolis rule [52]. Along the simulations and at regular time intervals, an exchange between neighboring copies at slightly different μ’s is attempted, and then accepted or rejected according to a probability that involves the energy cost of such a move. We note that in a grand canonical ensemble (μVT), the number of particles (per unit area) for each species, $c_{i}=L^{-2}\sum_{x}\rho_{i}\left(x\right)$, fluctuates around an average value at equilibrium prescribed by the μ’s.

The outcome of these parallel tempering runs will be the equilibrium average concentrations ${\bar{c}}_{i}\equiv \langle c_{i}\rangle$ of each species in the whole membrane as a function of the chemical potential, a quantity that is an analog to an adsorption isotherm. Also the fluctuations of the particles concentrations are considered here, since they provide access to the compressibility of the mixture κ(μ,T), defined as $\kappa =c_{1}\;\Delta c_{1}+c_{2}\;\Delta c_{2}$ with $\Delta c_{i}$ being the variance of the i-type particle number density, i.e., $\Delta c_{i}\equiv \langle {\left(c_{i}-{\bar{c}}_{i}\right)}^{2}\rangle$.

In addition to the particle concentration and its fluctuations, the energy of the system given by Equation (3) was also considered, together with the specific heat defined as $C_{v}\left(\mu ,T\right)\equiv \left(\langle E^{2}\rangle -{\langle E\rangle }^{2}\right)/L^{2}$, which provides a thermodynamic signature for possible phase transitions.

Finally, we also performed simulations in the canonical ensemble (NVT), aimed to describe the time evolution of the mixture at finite temperature and with conserved densities. Now, in addition to a constant temperature *T*, we also fixed the number of particles $N_{1},N_{2}$ of both species during the simulation. In this case, the Monte Carlo moves consist of displacements of a randomly chosen particle to an empty neighboring cell. These moves are then accepted or rejected following the Metropolis rule.

## 3. Results

### 3.1. The system in Equilibrium with a Reservoir of Particles: Ground State Calculations

**Figure 2:**
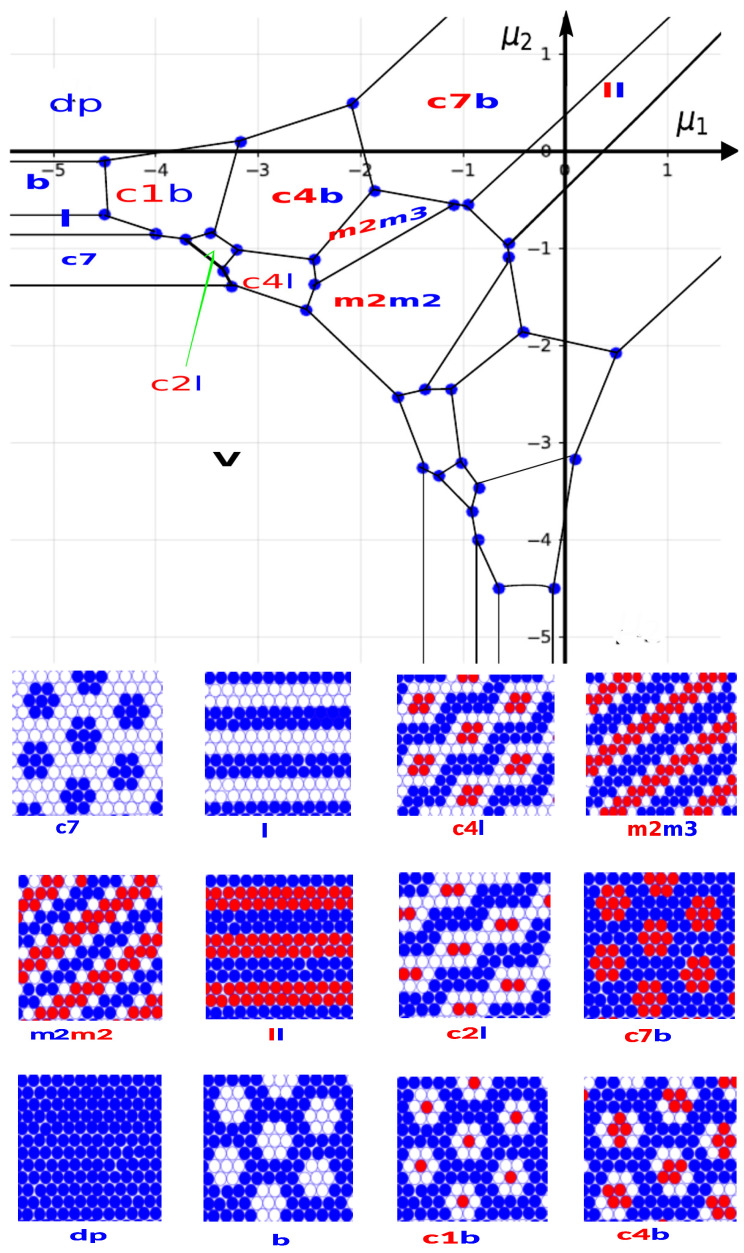
The ground state of the open system with patterns corresponding to the lowest energy for different parts of the plane of the chemical potentials of the two components, $\left(\mu_{1},\mu_{2}\right)$. Such ordered patterns are thermodynamically stable for T→0. The patterns are labeled according to the following scheme: ‘cn’—clusters made of n molecules; ‘l’—layers (or stripes); ‘mn’—modulated layers of thickness n; ‘b’—bubbles; ‘dp’—densely packed. Finally, ‘v’ denotes vacuum, i.e., all cells empty. Each pattern is characterized by a pair of symbols, except for ‘v’ and the patterns made by one-component molecules. The first symbol in the pair (in red) concerns the first component, and the second symbol in the pair (in blue) concerns the second component. Because of the symmetry of the model, the GS is symmetric, and the symmetry axis is $\mu_{2}=\mu_{1}$. The patterns for $\mu_{2}>\mu_{1}$ are shown in the graphs below the $\left(\mu_{1},\mu_{2}\right)$ diagram. For $\mu_{2}<\mu_{1}$, the patterns are the same as for $\mu_{2}>\mu_{1}$, but with interchanged components 1←→2. The patterns for $\mu_{2}<\mu_{1}$ can be obtained by changing the red circles to the blue ones, and vice versa.

In this section, we find the patterns that minimize the thermodynamic Hamiltonian (4) per lattice cell for given $\mu_{1},\mu_{2}$ by comparing the value of *H* per lattice cell for different periodic patterns. In practice, we considered a unit cell of each pattern, and assuming periodic boundary conditions, we calculated $h=H/A_{c}$, where $A_{c}$ is the number of lattice cells in the unit cell of the considered pattern. To illustrate the calculation of *H* on the simplest example, let us consider a single ‘flower’ cluster consisting of seven molecules of the second component (see the clusters in the c7 pattern in Figure 2), and assume that $\mu_{1}$ is so strongly negative that no proteins of the first component can appear. There are 12 pairs of nearest neighbors, and 3 pairs separated by |Δx|=2 in the cluster. Thus, from (1), (2), and (4), we obtain $H/J_{1}=-12+3J-7\mu_{2}$. Note that H=0 for $\mu_{2}=\left(3J-12\right)/7$. Thus, for this value of the chemical potential controlling the exchange of the membrane proteins with the bulk reservoir, different numbers (including 0) of well-separated ’flower’ clusters can appear in thermal equilibrium. On the other hand, an empty lattice is stable for very small $\mu_{1}$ and $\mu_{2}<\left(3J-12\right)/7$, whereas for $\mu_{2}>\left(3J-12\right)/7$, the c7 pattern minimizes *h*. Similar rather simple but tedious calculations allowed us to find the stable pattern for each region of the $\left(\mu_{1},\mu_{2}\right)$ diagram. It turns out that for J=3/4, not only is the size of the clusters larger than for J=3, but also many more ordered patterns can be stable. The ground state (GS) of the open system corresponding to the minimum of *h* is shown in Figure 2, together with the obtained ordered patterns.

In order to label different patterns, we usd the symbols ‘cn’, ‘l’, ‘mn’, and ‘b’ for clusters of n molecules, stripes (or lamellas), modulated stripes of thickness n, and bubbles in dense liquid, respectively. In addition, by ‘dp’ and ‘v’, we denote the densely packed (full occupancy of the lattice cells) and vacuum (empty lattice) phases, respectively. The patterns composed by the two types of the proteins will be identified by a pair of symbols. The first (red) and the second (blue) symbols in the pair will refer to the first and the second component, respectively. For example, c4b denotes clusters of four first-component molecules inside bubbles in the liquid of the second component, bc4 denotes clusters of four second-component molecules in bubbles in the liquid of the first component, m2m3 denotes modulated thinner and thicker stripes of the first and the second component, respectively, and ll denotes lamellas of both components. The patterns and the corresponding symbols are shown in Figure 2.

We compare the energetically favored patterns with the simulation snapshots for low but finite *T* in the next subsections.

### 3.2. Monte Carlo Simulations in the Grand Canonical Ensemble: Low-Temperature Ordering

We use the phase diagram at T=0 obtained by ground state calculations and shown in Figure 2 as a guide to explore the possible phase equilibria of the mixture at finite, low temperatures by means of Monte Carlo simulations of the lattice model in the grand canonical ensemble. Thus, we set the temperature at a constant value, $T^{*}=k_{B}T/J_{1}=0.1$, and scanned certain ranges of chemical potentials $\mu_{1},\mu_{2}$ that are representative of the different patterns displayed by the phase diagram of Figure 2.

We present below the main findings of the simulations for the specific cases studied.

#### 3.2.1. A mixture with Symmetric Composition ($\mu_{1}$ = $\mu_{2}$)

We started the survey on the low-temperature behavior of the mixture by considering the situation where both species had the same chemical potential ($\mu_{1}$ = $\mu_{2}$ = $\mu_{i}$). In terms of the $\mu_{i}$ variables, this is equivalent to studying the system along the diagonal of the phase diagram in Figure 2 and ensures that, on average, the number of particles will be the same for both types i=1,2.

Following the GS calculation, one can expect the vacuum phase to dominate at low enough chemical potentials $\mu_{i}$ < $\mu_{v}$ = −25/12, while for higher values, the presence of two ordered phases is predicted, each one displaying specific structural periodic motifs: the m2m2 phase in the range $\mu_{v}$ < $\mu_{i}$ < $\mu_{l}$ = −3/4 and the ll phase for $\mu_{l}$ < $\mu_{i}$. The first one consists of alternating modulated stripes of the component-1 and component-2 separated by vacuum (see m2m2 in Figure 2) and is reminiscent of the *zz*-phase (“zig-zag”) obtained for the case of strong repulsion *J* = 3.0 studied in Ref. [43]. The second one is made of alternating stripes of each species and with the same thickness (see ll in Figure 2), i.e., it is indeed a lamellar phase. This type of ordered lamellar patterns found at equal concentrations of both components is commonly found in other systems, like block copolymer mixtures or surfactant solutions.

**Figure 3:**
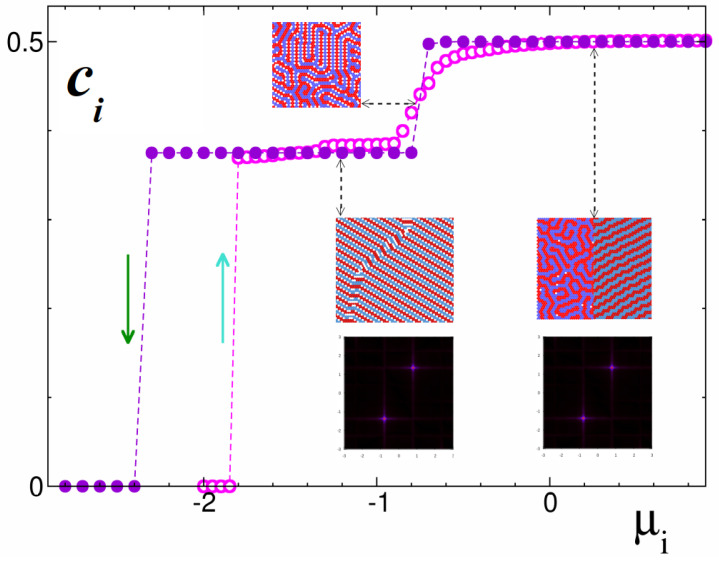
Grand canonical Monte Carlo simulations of a mixture with $\mu_{1}=\mu_{2}$, i.e., equal concentration for both components 1 and 2. Concentration profiles $c_{i}\left(\mu_{i}\right)$ for isotherms taken at $T^{*}=0.1$ and starting from a random distribution of particles (empty symbols) or an ordered configuration (full symbols). The chemical potentials are in units of the nearest-neighbor interaction $J_{1}$, and the dimensionless temperature is $T^{*}=k_{B}T/J_{1}$. The insets show typical configurations for three representative states, as indicated. The structure factor S($\bar{k}$) in the reciprocal space (in units of $1/a^{2}$ with *a* as the lattice constant) is included for both types of ordered patterns: m2m2 (left) and ll (right). For the equilibrium patterns and the corresponding symbols, see Figure 2.

The density dependencies on the chemical potential shown in Figure 3 were obtained from isotherms at $T^{*}=0.1$, starting with two different initial conditions, either a random distribution of both species (empty symbols) or an ordered m2m2 configuration (full symbols). At low chemical potential, we observe a sharp increase (decrease) in the particle density, respectively, occurring at rather different values of μ for each case. This hysteresis loop indicates the limits for the stability of both phases. In fact, for the m2m2 phase the location of the transition agrees well with the predicted value $\mu_{v}$. In a similar manner the transition to the lamellar phase at higher chemical potential is abrupt for the ordered configurations, but smooth for the disordered ones. In any case, however, it takes place at the predicted value $\mu_{l}$ = −3/4.

The representative configurations included in Figure 3 to illustrate the equilibrium patterns deserve a comment. Since we were simulating at finite temperatures, the presence of some type of defect is expected, especially for the runs started from disordered configurations. This is more clearly seen in the lamellar state for $\mu_{i}$ > 0, where the two snapshots included correspond to each of the starting states. Furthermore, the coexistence of both m2m2 and ll phases around $\mu_{l}$ is nicely illustrated by the configuration included in the upper part of the figure, where small domains of the ll phase are present between larger domains of the m2m2 phase.

The information provided by the structure factor S($\bar{k}$) also supports the picture of periodic order in the mixture with symmetric composition. Since the dispersion has its origin in the particles, the two spectra shown in the inset of Figure 3 display the same spot pattern. This is due to the fact that both phases have a periodicity *D* = 4 in the triangular lattice, whereas the only difference is that empty sites between stripes in the m2m2 phase are occupied randomly by particles of both species as one increases the chemical potential. Actually, the S($\bar{k}$) for these motifs resembles the one obtained for the former zz phase found in the *J* = 3.0 case.

#### 3.2.2. Mixtures with $\mu_{1}\neq \mu_{2}$

We consider now the case of mixtures containing different numbers of particles for each species in order to investigate the role of the composition on the phase equilibria at low temperatures. Since a detailed survey of the whole $\mu_{1},\mu_{2}$ plane would imply a huge simulation effort, we decided to explore a reduced set of parameters. These values were chosen after revising the GS results, trying to identify the most prominent among the different patterns that were predicted.

**Figure 4:**
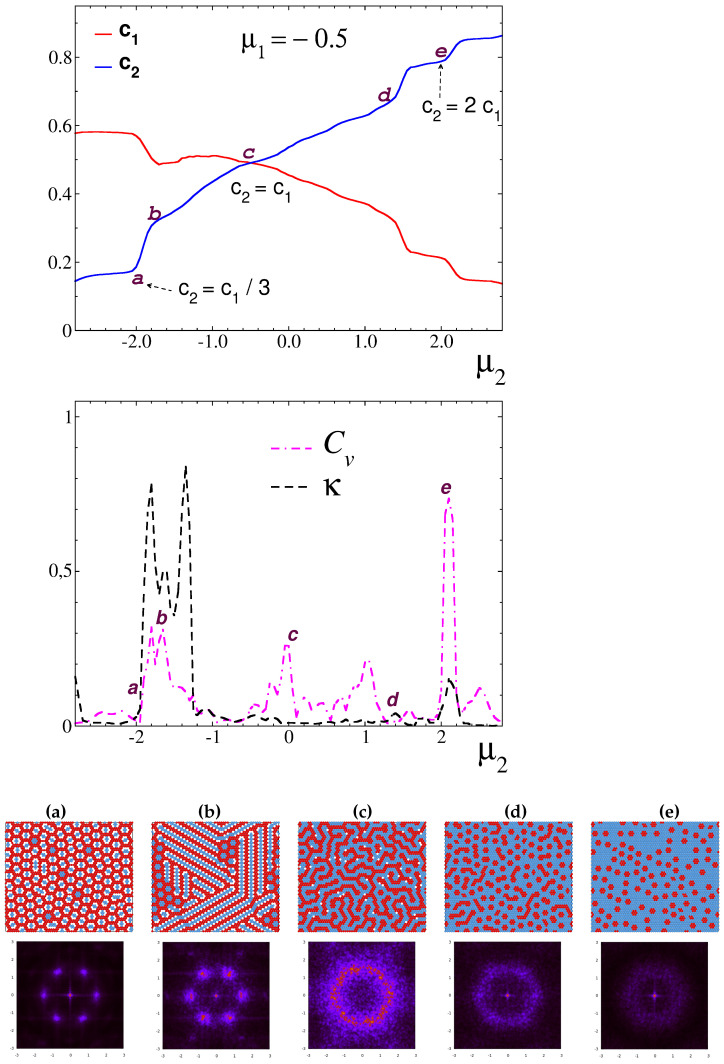
Grand canonical Monte Carlo simulations of a mixture with $\mu_{1}$ = −0.5. **Top**: concentration profiles $c_{i}$ for each of the species as a function of the chemical potential $\mu_{2}$, calculated at a temperature $T^{*}$ = 0.1. The chemical potentials are in units of the nearest-neighbor interaction $J_{1}$, and the dimensionless temperature is $T^{*}=k_{B}T/J_{1}$. **Middle**: specific heat $C_{v}$ and compressibility κ. Both quantities were rescaled for the sake of clarity. For definitions, see the text. **Bottom**: representative configurations and structure factor S($\bar{k}$) for the state points (**a**–**e**) indicated in the top figure, respectively. For a comparison of the patterns with the patterns stable at T→0, see Figure 2.

**The case of** $\mu_{1}$ = −0.5. We start with a fixed $\mu_{1}$ = −0.5 and explore the concentration and thermodynamic quantities across a broad range of the chemical potential $\mu_{2}$ at a fixed temperature $T^{*}$ = 0.1; see Figure 4. For low values $\mu_{2}$ < −5.0 the b1 phase is stable, and the lattice is populated by hexagons made of seven empty sites. These bubbles, in turn, exhibit a trend to organize themselves into a regular lattice (see the bubble pattern in Figure 2 with the blue circles replaced with the red ones), much in the same way as hard disks in the continuum two-dimensional space. Interestingly, the average density of type-1 particles is now $c_{1}\approx 0.6$, just above the ratio 7/12 between the number of empty sites in a hexagon (7) and the number of type-1 particles in the row surrounding it (12). Nevertheless, the long-range ordering of these *soft* bubbles into a regular hexagonal pattern is not fully developed because of commensurability effects on the lattice.

For −5.0 < $\mu_{2}$ < −2.0, the empty hexagons start to be occupied at their centers by the type-2 particles that gradually appear. At the same time, some of these hexagons merge themselves to create larger bubbles that have a regular, slightly elongated shape resulting from the (partial) overlap of two or more hexagons. These voids are then gradually occupied by clusters of type-2 particles, mostly with a rhomboidal shape, displaying a c4l phase; see snapshot (a) in Figure 4. Then, at the value $\mu_{1}\approx$−2.0, the mixture undergoes a transition to the m2m3 phase, as shown by a steep decrease/increase in the concentrations $c_{1}$/$c_{2}$, respectively. This phase change is accompanied by a peak in both the specific heat $c_{v}$ and the compressibility κ. We note that even though the observed jump in the concentrations $c_{1}$ and $c_{2}$ at the *a → b* transition, and the presence of a peak in the specific heat in Figure 4 suggest that it is a first-order transition, a definite answer to this issue requires a careful analysis of the finite-size effects affecting the simulation results. This task, however, is out of the scope of the present work.

When the chemical potentials of both species are similar, the system exhibits the expected lamellar pattern, as shown in snapshot (c) in Figure 4, where the molten lamella has a thickness of 4, and the overall density is ρ≈ 1. These structures become unstable and begin to deaggregate as type-2 is the majority component, and now, both the hexagonal clusters and the stripes of variable length and orientation are observed coexisting on the membrane.

The observed progression of different types of order is confirmed by the calculated structure factor shown at the bottom of Figure 4, which reflects either the presence or the absence of periodicity. For example, in the case where any of the species span the lattice, S($\bar{k}$) will show a spot at the origin $\bar{k}$ = 0, while the typical six-fold dispersion pattern denotes the periodic ordering of particles along the lattice axes. The molten lamella, on the other hand, exhibits a rather smeared pattern due to a broad distribution of lengths and orientations of the stripes. Finally, as soon as the hexagonal clusters are immersed in a sea of the other component and distribute themselves randomly, the most prominent spot in S($\bar{k}$) is the one at $\bar{k}$ = 0.

**Figure 5:**
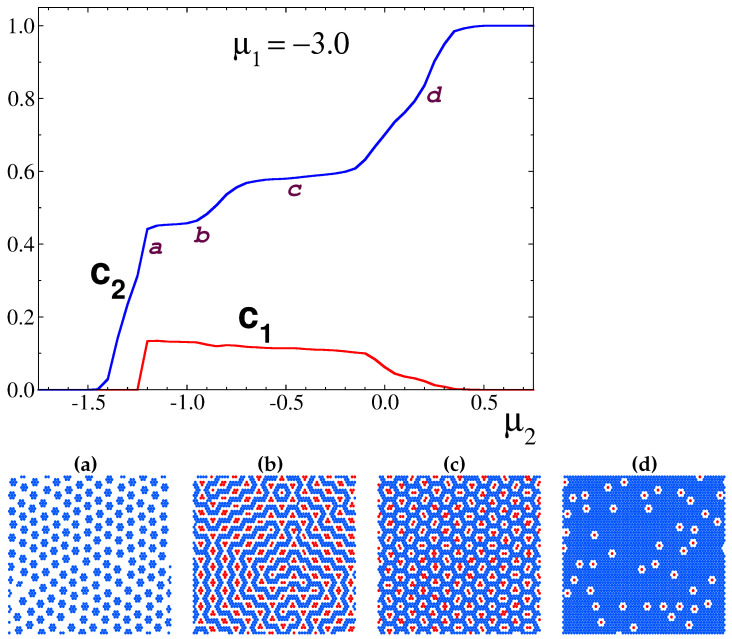
Grand canonical Monte Carlo simulations of a mixture with $\mu_{1}$ = −3.0. **Top**: concentration profiles $c_{i}$ for each of the species as a function of the chemical potential $\mu_{2}$, calculated at a temperature $T^{*}$ = 0.1. The chemical potentials are in units of the nearest-neighbor interaction $J_{1}$, and the dimensionless temperature is $T^{*}=k_{B}T/J_{1}$. **Bottom**: structural evolution upon increasing $\mu_{2}$ for the state points (**a**–**d**) indicated above, respectively. For a comparison of the patterns with the patterns stable at T→0, see Figure 2.

**The case of** $\mu_{1}$ = −3.0. Upon further increasing the asymmetry in the number of particles for each species, we found even more different ordering patterns. We then set $\mu_{1}$ = −3.0 and again performed a scan of the chemical potential of the second component at a fixed temperature $T^{*}$ = 1.0. Starting from a low value of $\mu_{2}$ (but with $\mu_{1}$ < $\mu_{2}$), the lattice becomes increasingly covered by type-2 particles that aggregate to form hexagonal clusters; see Figure 5. These clusters, in turn, organize themselves in a hexagonal order (the c7 phase) that is destroyed once $\mu_{2}$ reaches a value of −1.25. Now, the rather small concentration of type-1 particles allows for the formation of the c4l phase that combines connected stripes of the majority component with tetragonal clusters of the minority species. The stripes coalesce as $\mu_{2}$ increases further and a pattern made of soft bubbles emerges. These bubbles mostly adopt the shape of two overlapped hexagons and appear to be filled by tetragonal clusters of type-1 particles (i.e., the c4b pattern). However, some variation in the shape and the size of the bubbles can be observed in the snapshots, where larger triangular voids filled by a variable number of type-1 particles are also present.

For $\mu_{2}\geq 0$, the concentration of type-2 particles increases, while that of type-1 particles continuously decreases in such a way that the c4b pattern gives rise to a diluted phase of hexagonal bubbles filled by only one type-1 particles, i.e., the c1b phase. The progressive increase in $c_{2}$ and decrease in $c_{1}$ makes these bubbles unstable and a dense type-2 phase is eventually formed.

**Figure 6:**
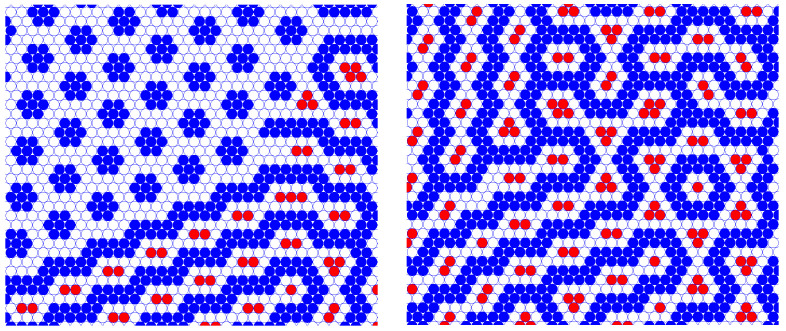
Snapshots of GCMC simulations at $T^{*}=0.1$ and $\mu_{1}=-3.4,\mu_{2}=-1$ showing the c2l phase in coexistence with the c7 phase (**left**) and at $\mu_{1}=-3.3,\mu_{2}=-1$ with the domains of the c2l phase with different orientations and inclusions of more complex motifs (**right**). The chemical potentials are in units of the nearest-neighbor interaction $J_{1}$, and the dimensionless temperature is $T^{*}=k_{B}T/J_{1}$. For a comparison of the patterns with the patterns stable at T→0, see Figure 2.

We should note that the progression of the patterns for $\mu_{1}$ = −3.0 and increasing $\mu_{2}$ in the GS (Figure 2) is different from the above-described sequence of patterns. In particular, the c7 motif is present on the GS (Figure 2) for $\mu_{1}$ < −3.0. This is because the phase coexistence lines at $T^{*}=0.1$ are slightly shifted compared to the lines at $T^{*}=0$ due to thermal motion. The patterns present in simulations for $T^{*}\;=\;0.1$ and $\mu_{1}$ = −3.0 appear on the GS for −3.5 < $\mu_{1}$ < −3.0. In addition, the c2l pattern present on the GS was observed in simulations for $\mu_{2}$ = −1.0 and $\mu_{1}$ = −3.3, −3.4 (see Figure 6), and a trace of it can be seen in snapshot (b) in Figure 5.

### 3.3. Monte Carlo Simulations with a Fixed Number of Particles

In this section, we explore the kinetics of ordering and self-assembling processes occurring in the mixture at finite temperature and constant densities. We performed simulations in the canonical ensemble with a fixed number of particles, and the time evolution of the system proceeded through the simple diffusion of both types of particles across the lattice. The displacement of a randomly chosen particle takes place to a first neighbor site (also chosen at random between the available empty sites), and the proposed move is accepted with a probability $p∼\Delta E/k_{B}T$, where ΔE is the change in energy produced by the displacement.

We kept the total number of particles fixed at *N* = $N_{1}$ + $N_{2}$ = 780 and started with a randomly distributed configuration at a relatively high temperature $T^{*}$ = 2.0. Then, we slowly cooled down the system toward a final low temperature $T^{*}$ = 0.125. This procedure ensures that the system is driven through successive equilibrium states all along the thermal relaxation.

**Figure 7:**
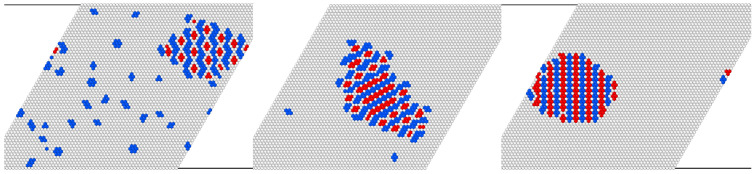
Monte Carlo simulations of mixtures with a fixed number of particles, *N* = $N_{1}$ + $N_{2}$. Representative configurations for low-temperature ordering. The overall number density on the lattice is kept fixed at ρ = 0.1, and the ratios η of type-1 to type-2 particles are 0.2, 0.5, and 1.0, respectively.

We followed the evolution of the assembling process through a visual inspection of the instantaneous configurations in order to check for the formation of any kind of ordered patterns. Here, we only show snapshots that correspond to the final state that was reached by the mixture for each of the compositions studied. In Figure 7, we compile the results for the case of a density ρ = $N/L^{2}$ = 0.1 and three different ratios of type-1 to type-2 particles (η = $N_{1}$/$N_{2}$). With this rather low coverage of the lattice, we expect to mimic the situation where the mixture is in equilibrium with the vacuum and expect the presence of ordered patterns. The snapshots in Figure 7 actually confirm these expectations. For a composition η = 0.2, we observe the appearance of a large domain of the c4l phase that coexists with some hexagons that correspond to the c7 phase. Also, a few other clusters of variable shape and size (although small) are present. Upon increasing the number of type-1 particles, the system displays the coexistence of the c4l motif with the m2m2 phase. Finally, with a higher ratio η = 1.0 (i.e., an equal number of type-1 and type-2 particles), a large domain with the m2m2 pattern is obtained.

## 4. Summary and Conclusions

We put forward a hypothesis that the further growth and coalescence of the clusters of membrane proteins are inhibited by underscreened electrostatic interactions. The hypothesis is based on the recent experimental discovery of long-range forces between charged objects in concentrated electrolytes [19,20,21,22] confirmed by simulations and theory [23,24,25,26,27,28,29,30]. Indeed, at large separations the electrostatic repulsion can dominate over the Casimir attraction associated with critical concentration fluctuations, if the latter is stronger but of a shorter range. On the other hand, the Casimir attraction dominates at short distances. Such effective short-range attraction–long-range repulsion (SALR) interactions can lead to the formation of well-separated clusters with optimal size if the number of proteins is not too large.

If the above hypothesis is correct, then the distribution of the membrane proteins can be governed by the competition between the Casimir and the electrostatic potentials. To study the outcomes of this competition on a very general level, we focused on a question of energetically favorable patterns in a binary mixture with the SALR potential between like particles and the cross-interaction of the opposite sign. To this end, we considered a triangular lattice model and focused on two cases. In the first case, the membrane proteins are in equilibrium with a reservoir characterized by the chemical potentials $\mu_{1},\mu_{2}$ that control the density of the proteins. In the second case, the numbers of the proteins are fixed.

We found that the energetically favorable patterns depend significantly on the shape of the SALR potential, especially on the relative strength of its repulsive and attractive parts, and on the density and proportions of the membrane proteins. In our case of the repulsion to attraction ratio J=3/4, there are many more ordered patterns, and the ground state $\left(\mu_{1},\mu_{2}\right)$ is more complex than for the J=3.0 studied in Ref. [43]. This is in contrast to one-component SALR systems, where changes in the shape of the potential lead only to change in the size of the assemblies.

For a fixed number of particles, dispersed clusters are formed when the second component is absent. The addition of a small amount of the second component leads to the formation of a single two-component cluster with the proteins forming a regular pattern. The number of dispersed first-component clusters decreases, and the pattern in the two-component cluster changes with the increasing density of the second component. For comparable amounts of the two components, all particles are involved in the big single cluster (Figure 7). Thus, the second component can work as a trap for the small clusters of the first component.

We conclude that the two universal competing forces can lead to surprisingly rich pattern formation in quasi-2D mixtures. The patterns can be controlled by the shape of the SALR potential, by equilibrium with the bulk reservoir, or by the numbers of the macromolecules in the membrane. Thermal motion destroys the long-range order, but a short-range partial order remains and is even more interesting, as shown by the simulation snapshots in Figure 3, Figure 4, Figure 5 and Figure 6.

## Data Availability

The data supporting the conclusions of this article will be made available by the authors upon reasonable request.
